# Nitrous Oxide for Labor Analgesia: What We Know to Date

**DOI:** 10.31486/toj.19.0102

**Published:** 2020

**Authors:** Kirbie Broughton, Allison G. Clark, Adrienne P. Ray

**Affiliations:** ^1^ Department of Anesthesiology, Ochsner Clinic Foundation, New Orleans, LA; ^2^ The University of Queensland Faculty of Medicine, Ochsner Clinical School, New Orleans, LA

**Keywords:** *Analgesics*, *labor–obstetric*, *labor pain*, *nitrous oxide*

## Abstract

**Background:** Although nitrous oxide (N_2_O) has been used since the 1880s for labor analgesia, its popularity has only recently increased in the United States. In 2011, only 3 centers in the country offered N_2_O, but as of 2020, several hundred labor units have adopted its use.

**Methods:** We reviewed the literature and summarize the mechanism of action, clinical uses, and efficacy of N_2_O for labor analgesia, as well as patient satisfaction related to its use.

**Results:** N_2_O has several proposed mechanisms of action that make it a viable option for all 3 stages of labor and postpartum procedures. N_2_O has been shown to be a safe option for both mom and baby during labor and delivery. Studies support N_2_O as an analgesic for laboring. Even though 40% to 60% of women who use N_2_O convert to a labor epidural analgesia, satisfaction surveys indicate that analgesia is not the only factor contributing to the use of N_2_O during labor.

**Conclusion:** The use of N_2_O has increased in labor and delivery units across the United States since 2011. Despite inferior analgesic properties compared to epidural analgesia, N_2_O offers a safe alternative for many parturients who want a greater sense of control and mobility.

## INTRODUCTION

Women in the United States have fewer options for pain management than women in many other developed countries.^1^ Most hospitals in the United States offer neuraxial analgesia or intravenous opioids for pain relief during labor. However, many countries have other pain management strategies available such as hypnosis, acupuncture, and electrical stimulation. Most of these options are not routinely offered in the United States.^1^ Nitrous oxide (N_2_O) has long been used for labor analgesia in some centers, but only since 2011 has it grown in popularity in the United States. While neuraxial techniques are the most effective current option for pain control,^2^ N_2_O has been shown to provide analgesic properties.^3,4^

In this review, we discuss the history and growing popularity of N_2_O, its mechanism of action, maternal and fetal safety, efficacy, and satisfaction when used for labor analgesia.

## HISTORY

The discovery of N_2_O is attributed to English scientist Joseph Priestley in 1772; however, the first reported use of N_2_O for vaginal delivery did not occur until 1881.^5^ Polish physician Stanislav Klikovich used an 80% N_2_O/20% oxygen (O_2_) mixture in 25 laboring women and found that it provided analgesia without adverse fetal effects.^1^ The utility of N_2_O progressed during the early 20th century; however, its use was not without challenges. The lack of a systematic device to administer N_2_O and a way to ensure the delivery of safe concentrations were the two greatest limitations to the use of N_2_O in obstetrics. British anesthetist Robert James Minnitt was a pioneer of inhalational analgesia. Minnitt, along with instrument maker Charles King, produced the first gas/air apparatus in 1933. This device allowed women in labor to inhale a mixture of nitrous and air, providing one of the only forms of labor analgesia at the time. After being used for approximately 3 decades, this apparatus was withdrawn because of delivery of the hypoxic mixture of N_2_O and air.^6^ After multiple revisions to the apparatus, the British began using an N_2_O/O_2_ delivery system—named Entonox—in 1961.^7^ As of 2011, only 3 US academic centers were using N_2_O for labor analgesia. In 2011, Nitronox (Porter Instrument) became the first US Food and Drug Administration–approved N_2_O delivery system, which is set to deliver a fixed concentration of 50% N_2_0/50% O_2_. According to a 2012 review by Collins et al, the University of California, San Francisco, was at the forefront of institutions offering N_2_O for labor analgesia, at that point having offered the option for more than 30 years.^1^ Vanderbilt University and the University of Washington were the next large academic centers to offer N_2_O to parturients, and many other centers have followed. By 2018, more than 500 birthing centers and hospitals across the country had adopted N_2_O use.^8,9^

## MECHANISM OF ACTION

N_2_O is a tasteless, odorless vapor with peak brain concentrations occurring within 60 seconds after the onset of administration.^10^ Among the several proposed mechanisms for the pharmacokinetics of N_2_O are (1) N-methyl-D-aspartate antagonism, (2) pain perception modulation at the alpha-2 receptors in the dorsal horn of the spinal cord, and (3) the release of endogenous opioids in the brain. These various mechanisms are believed to reduce pain sensitivity and provide analgesia; however, these mechanisms are still not well understood. Despite insufficient evidence on the precise mechanism of action, N_2_O used for labor analgesia has not been associated with any major side effects and is generally well tolerated. However, as with any treatment therapy, N_2_O is associated with adverse reactions and has contraindications to its use. Nausea, vomiting, and dizziness are among the most common maternal side effects, reported by up to 46%, 14%, and 23% of parturients, respectively.^7^ Respiratory depression is rare unless N_2_O is used in the setting of systemic opioids.

With regard to neonatal outcomes, no significant adverse effects have been reported.^3,11^ Stefani and colleagues found no differences in neurobehavioral assessments of neonates of mothers receiving nitrous concentrations of 30% to 50%.^11^ Concerns about neurotoxic effects associated with N_2_O were raised when studies on the effects of anesthetics showed neuronal apoptosis in rodents; however, N_2_O by itself caused little or no apoptosis in the infant rat brain.^12^ Likis and colleagues analyzed 29 studies reporting fetal or neonatal outcomes based on umbilical cord gases and Apgar scores and found no significant difference between mothers who received N_2_O during labor vs mothers who did not.^3^

Contraindications to the use of N_2_O are limited and principally include administration to patients at risk for its accumulation in enclosed spaces (ie, pneumothorax, small bowel obstruction). Parturients with congenital heart defects and/or pulmonary hypertension should avoid N_2_O because it increases pulmonary vascular resistance.

Controversy regarding environmental exposure to N_2_O among healthcare workers and its association with increased risk of adverse reproductive outcomes has been ongoing. The use of scavenger systems and vigilant monitoring of exposure levels with dosimetry badges can keep exposure below the National Institute for Occupational Safety and Health (NIOSH) limit of 25 ppm. Long-term effects of N_2_O exposure are unclear; however, compliance with NIOSH standards is not associated with an increased risk of reproductive complications.^1,13^

## CLINICAL USE

N_2_O can be used for analgesia during the first, second, or third stage of labor; during postpartum procedures (eg, laceration repair, manual extraction of placenta); and to facilitate epidural placement. N_2_O is self-administered via a mask without straps that could keep the mask fixed to the face and potentially lead to excessive drowsiness and/or hypoxia. Studies evaluating the benefits of N_2_O state that the self-administration aspect of N_2_O improves the parturient's sense of control and ability to cope with labor.^5,9^ Some women do not like to be confined to a bed throughout their labor. The majority of neuraxial anesthetics cause weakness of the lower extremities, requiring a patient to remain in bed once the anesthetic is administered. While most N_2_O policies require the patient to remain in bed or a chair during use, the effects dissipate in less than 5 minutes. This short half-life gives the patient freedom to move about when not using N_2_O.^14^ Administration begins with the patient's inhalation that triggers the opening of a negative pressure valve and permits the flow of N_2_O. Educating the patient and practicing the correct technique with the patient is critical for successful pain relief.^9^ Because the onset of N_2_O is approximately 30 to 50 seconds, it should be initiated prior to the onset of a contraction so that peak serum concentrations are present at the height of the contraction.

## EFFICACY AND SATISFACTION

Varying results regarding the effectiveness of N_2_O for labor analgesia have been reported.^3,4^ A 2002 systematic review of 11 trials examining the efficacy of N_2_O revealed the complexity of this assessment.^4^ Several factors are responsible for the variability of findings: concentration of N_2_O used, method of administration, administration of other intravenous medications, and unsatisfactory study design. Because of this variability, drawing conclusions about the efficacy of N_2_O is difficult. In a postpartum survey of 2,482 Swedish women, 84% of nulliparous and 72% of parous women rated neuraxial techniques as very effective compared to 38% of nulliparous and 49% of parous women who rated N_2_O as very effective.^15^ Despite the lower percentage of satisfied parturients who used N_2_O, the study provides evidence that N_2_O has analgesic benefit. In a study comparing various analgesic modalities—including N_2_O, epidural alone, and epidural following other modalities (eg, N_2_O, meperidine)—33% of patients who used nitrous alone rated its analgesia as good.^16^ Subsequent studies continued to show a wide range of analgesic efficacy.^3,9^ Importantly, based on postpartum surveys of labor and delivery experience, analgesia is not the only factor associated with satisfaction. Other factors such as bodily sensations of labor, mobility, and perceived situational control are also major determinants of labor and delivery experience.^9^

## PREDICTORS OF SUCCESS

Sutton et al retrospectively analyzed the use of N_2_O and predictors of conversion to epidural labor analgesia.^17^ Three percent of parturients used N_2_O during the study period; the majority were nulliparous (71.2%), and more than half (51.9%) expressed preference for a nonmedical birth. Of the 146 women included in the analysis, 63% converted from N_2_O to neuraxial analgesia. Factors associated with conversion from N_2_O to neuraxial analgesia were labor induction, labor augmentation, and lower cervical dilation at the time of N_2_O request.^17^ Richardson et al analyzed qualitative comments from women who used N_2_O for labor to try to better understand the determinants of satisfaction despite variable analgesic effects. Of the 264 women who responded, 90% were highly satisfied with their use of N_2_O for labor. Many of these women reported coping benefits of N_2_O, such as relaxation, reduced anxiety, and dissociation from pain. Richardson et al reported that among patients who used N_2_O and delivered vaginally, 40% converted to neuraxial analgesia.^9^

## CONCLUSION

Despite its long history, N_2_O is a relatively new option for labor analgesia in the United States. While the mechanism of action of N_2_O is unclear and pain relief does not compare to the efficacy of neuraxial techniques, evidence shows that N_2_O provides analgesic benefit in labor. The many advances made in the N_2_O delivery system ensure that parturients receive safe concentrations, and, despite common side effects, no serious adverse effects have been reported. Despite some studies reporting suboptimal pain control, N_2_O offers situational control, mobility, and bodily sensation in labor that are associated with greater satisfaction among some parturients. The increasing availability of N_2_O shows promise for improved management of labor pain for women and opens the door for further advancement in options available to laboring parturients.

## Notes

This article meets the Accreditation Council for Graduate Medical Education and the American Board of Medical Specialties Maintenance of Certification competencies for Patient Care and Medical Knowledge.
